# Development and validation of lumbar spine finite element model

**DOI:** 10.7717/peerj.15805

**Published:** 2023-08-11

**Authors:** Tomasz Wiczenbach, Lukasz Pachocki, Karol Daszkiewicz, Piotr Łuczkiewicz, Wojciech Witkowski

**Affiliations:** 1 Department of Mechanics of Materials and Structures, Faculty of Civil and Environmental Engineering, Gdańsk University of Technology, Gdańsk, Pomerania, Poland; 2 2nd Division of Orthopedics & Kinetic Organ Traumatology, Faculty of Medicine, Medical University of Gdańsk, Gdańsk, Pomerania, Poland

**Keywords:** Numerical modeling, FEM, Lumbar spine, Validation, Biomechanics

## Abstract

The functional biomechanics of the lumbar spine have been better understood by finite element method (FEM) simulations. However, there are still areas where the behavior of soft tissues can be better modeled or described in a different way. The purpose of this research is to develop and validate a lumbar spine section intended for biomechanical research. A FE model of the 50th percentile adult male (AM) Total Human Model for Safety (THUMS) v6.1 was used to implement the modifications. The main modifications were to apply orthotropic material properties and nonlinear stress-strain behavior for ligaments, hyperelastic material properties for annulus fibrosus and nucleus pulposus, and the specific content of collagenous fibers in the annulus fibrosus ground substance. Additionally, a separation of the nucleus pulposus from surrounding bones and tissues was implemented. The FE model was subjected to different loading modes, in which intervertebral rotations and disc pressures were calculated. Loading modes contained different forces and moments acting on the lumbar section: axial forces (compression and tension), shear forces, pure moments, and combined loading modes of axial forces and pure moments. The obtained ranges of motion from the modified numerical model agreed with experimental data for all loading modes. Moreover, intradiscal pressure validation for the modified model presented a good agreement with the data available from the literature. This study demonstrated the modifications of the THUMS v6.1 model and validated the obtained numerical results with existing literature in the sub-injurious range. By applying the proposed changes, it is possible to better model the behavior of the human lumbar section under various loads and moments.

## Introduction

The finite element method (FEM) has been widely used to improve road safety (John et al., 2022; Fang, Wang & Weggel, 2015; Sybilski & Małachowski, 2021; Bruski et al., 2019; Jones et al., 2016; Mendoza-Vazquez, Davidsson & Brolin, 2015). Simulations allow for investigating the behavior of the human body during a road accident. This feature offers the possibility of estimating the risk of fractures or damage to human soft tissues. One of the road traffic injuries with the most serious long-term consequences is a lumbar spine section injury (Zheng, Tang & Hu, 2018; Muller et al., 2014; El-Rich et al., 2009; Fradet et al., 2014; Eberlein, Holzapfel & Frohlich, 2004). Several new field studies have shown that lumbar spine fractures occur more frequently in new vehicle models than in previous models (Kaufman et al., 2013; Muller et al., 2014; Pintar et al., 2014; Doud et al., 2015). It is important to have reliable numerical models to explain this phenomenon.

Present crash test dummies were developed to assess injury criteria for the lower extremities, chest, neck, and head (Zheng, Tang & Hu, 2018). Their lumbar spine section arrangement was not based on actual human anatomy. Moreover, the validation for this section was not performed against any cadaver or human impact responses (Amiri, Naserkhaki & Parnianpour, 2020). Subsequently, despite the loading modes acting on the lumbar spine section being assessed in crash tests, they might not certainly follow the actual injury risk or fracture mechanism in frontal car crashes. Complete human body models, such as the Global Human Body Models Consortium (GHBMC), Virtual Vehicle Safety Assessment (ViVA), or Total Human Model for Safety (THUMS) are used to assess the risk of injury. Their advantage is a comprehensive approach to modeling, where the interaction of all body parts is taken into account. Moreover, they are usually validated against different experiments (Dreischarf et al., 2011; Guan et al., 2007; Heuer et al., 2008; Nachemson, 1960). The problem in their use may be the trade-off between the computation time and the level of complexity and discretization of a finite element (FE) model. This makes it problematic to study local phenomena by accurately identifying the location of failures. For this reason subsystems from a numerical model of the human body are used, such as models of the lumbar spine section. This allows for a more detailed analysis of the phenomena occurring in specific spine sections.

Various models of the lumbar spine can be found in the literature (Dreischarf et al., 2011; Xu et al., 2017; Schmidt et al., 2006; Renner et al., 2007; Wagnac et al., 2011). Before the model can be considered reliable, it is necessary to validate it against experimental results. Such validation has been described in previous works, such as in Zheng, Tang & Hu (2018); Östh et al. (2016); Wilke et al. (2001); Xu et al. (2017). Kiapour et al. (2012a) created a numerical model of the L3-S1 section based on computed tomography scans. This model has been validated using literature data for various load combinations (Goel et al., 2005; Kiapour & Goel, 2010; Kiapour et al., 2012b). The authors added a compression force to the model to simulate the weight of the upper body and muscles. A numerical model was used to analyze the movement of the lumbar spine after fusion surgery. Additionally, this model predicted the biomechanics of the spine after the insertion of an intervertebral disc implant. Zheng, Tang & Hu (2018) examined the lumbar spine injury mechanism in frontal car crashes. The effects of modification of thickness, stiffness, and angle of cushion, impact velocity, and coefficient of friction on lumbar spine injuries were analyzed. An article by Ayturk & Puttlitz (2011) presented several validation tests and indicated the need to extend the validation beyond the range of motion. Dreischarf et al. (2014) compared the results for several different numerical models that were considered validated. Thanks to the contribution of these works, it is possible to create a wider database of validated numerical models. This could potentially be used in the future to conduct statistical analyses, *e.g.*, by examining the influence of various factors on the susceptibility of the spine to injuries. Due to the extensive biodiversity of geometry and material characteristics of the human body and still a limited amount of validation data, it is essential to fully share the results that can be obtained with the FEM.

In this study, a subsystem of the 50th percentile adult male (AM) THUMS v6.1 model (Toyota Motor Corporation & Toyota Central R and D Labs. Inc., 2021) was used, *i.e.*, its lumbar spine section. Too stiff response of the THUMS lumbar spine model was observed in the flexion validation test for quasi-static and dynamic loading (see Fig. 4-44 and Fig. 4-46 in Toyota Motor Corporation & Toyota Central R and D Labs. Inc., 2021). Hence, the aim of the article is to improve the mechanical behaviour of the lumbar spine model in flexion and other validation tests. In order to achieve this goal nonlinear orthotropic material model was used for ligaments and modifications were introduced to the models of intervertebral discs to better reflect their anatomical structure and properties. Material parameters were taken from the literature and calibrated with respect to experimental flexion and compression tests, as they are of key importance in the analysis of road accidents (Richards et al., 2006; Ivancic, 2013; Pachocki et al., 2021; Tushak et al., 2023). In the present study, the set of validation tests were extended in relation the THUMS documentation (Toyota Motor Corporation & Toyota Central R and D Labs. Inc., 2021) by testing the range of motion in combined loading modes and testing the pressure level in the discs.

## Materials and Methods

**Figure 1:**
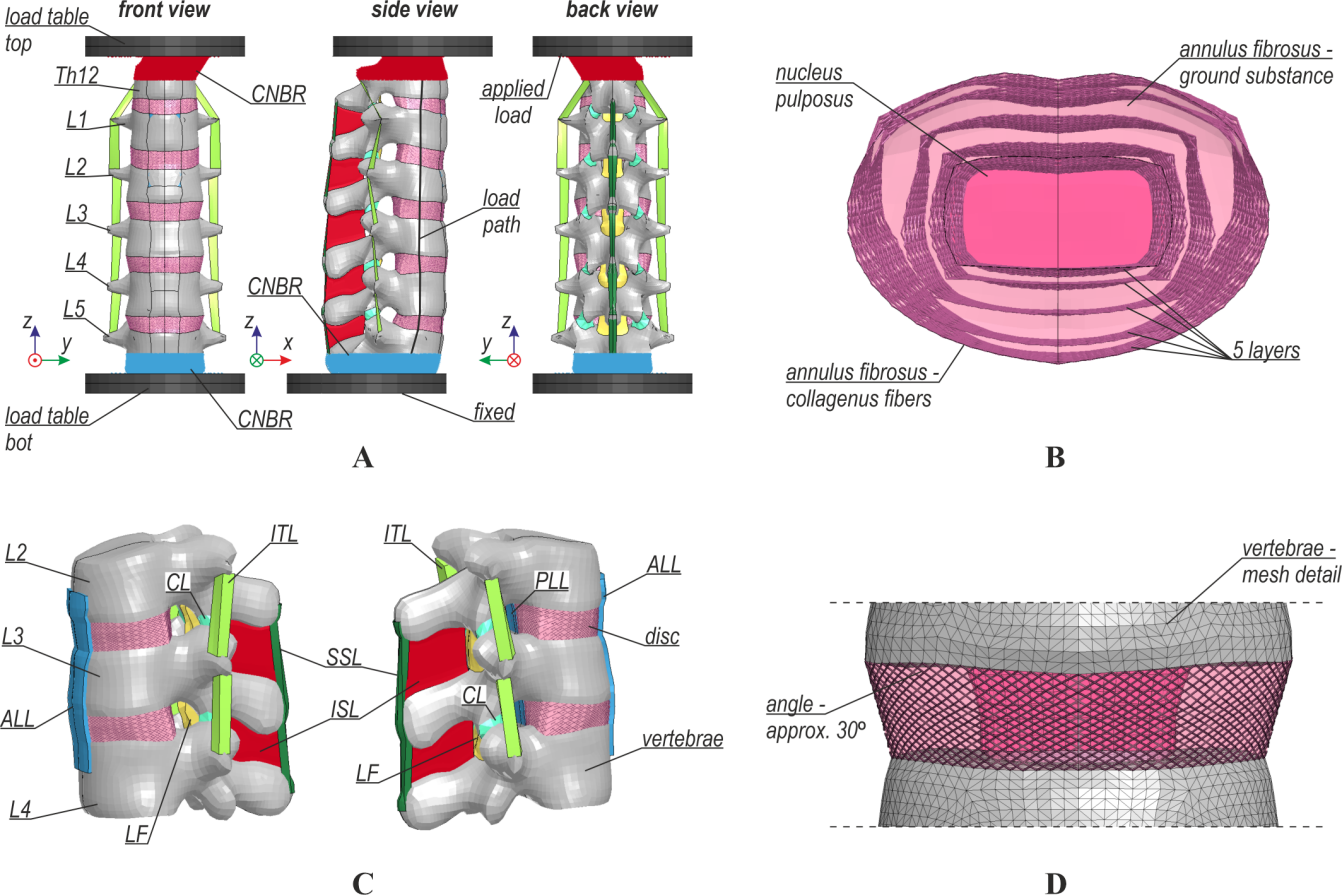
Numerical model: (A) general view, (B) structure of intervertebral disc, (C) ligaments, (D) intervertebral disc fibers.

In this study, a three-dimensional (3D) FE model was used to simulate the biomechanics of the lumbar spine. The model was based on the 50th percentile AM THUMS v6.1, developed by Toyota Motor Corporation and Toyota R&D Labs Inc. (Toyota Motor Corporation & Toyota Central R and D Labs. Inc., 2021). The lumbar spine of the THUMS was extracted, modified, and subjected to various validation procedures. The modifications were applied both to the geometrical and material properties of the model. The FE mesh was refined; it consisted of 14,818 and 111,458 nodes that comprised 9,435 and 37,740 shell FEs, 49,972 and 503,712 solid FEs, and 11 and 17,478 beams for the THUMS and the current model, respectively. Nonlinear explicit dynamic analyses were performed for the entire system using LS-DYNA software (Hallquist, 2006; LSTC, 2017a; LSTC, 2017b). The general view of the model is depicted in Fig. 1A.

### Intervertebral discs

The intervertebral disc (IVD) in the models consisted of the annulus fibrosus (AF) and nucleus pulposus (NP), as shown in Fig. 1B. The AF was a composite of collagenous fibers and ground substance. In the current model, nonlinear truss elements (*SECTION_SEATBELT) were used for the fibers, and 8-node solids (*SECTION_SOLID) with reduced integration (RI) were used for the ground substance. The interaction between the fibers and ground substance was enforced by shared nodes of their corresponding elements. The same RI solid formulation (*SECTION_SOLID) was applied for the NP in both models. The IVD model was adapted according to the literature (Shirazi-Adl, Ahmed & Shrivastava, 1986; Schmidt et al., 2006; Schmidt et al., 2007). After modifications, the content of collagenous fibers was 16% of the volume of the AF ground substance. Fibers were rearranged into five layers in a crisscross pattern with an angle of approximately 30° (see Fig. 1D). The distribution of the fibers was also modified so that the fibers on the outer layer had the greatest stiffness and cross-sectional area (*MAT_SEATBELT), and the innermost fiber layer had the lowest of these values (Shirazi-Adl, Ahmed & Shrivastava, 1986). The material properties of the ground substances of AF and NP were also modified. Their material law was changed to Mooney-Rivlin hyperelastic (*MAT_MOONEY-RIVLIN_RUBBER), and their mechanical properties were chosen based on data from the literature (Schmidt et al., 2006; Schmidt et al., 2007). For the AF, different values of material constants were assumed in three groups: L1-2, L2-4, and L4-5. The material properties of the IVDs are summarized in Table 1. Another modification was the separation of the NP from surrounding bones and tissues. Frictionless penalty-based contact was defined between the NP and the endplates and the surrounding AF.

**Table 1:** Material properties of the lumbar spine model.

**Structure name**	**Modulus, MPa**	**Density, Mg/mm^3^**	**Poisson’s Ratio, -**	**FE type**	**Material law**	**Ref.**
Cortical bone	13020	2.0e−9	0.3	4-node RI, nodal pressure avg	Piecewise linear plasticity with damage	THUMS v6.1
Cancellous bone	40	1.0e−9	0.2	8-node, FI solid	Piecewise linear plasticity with damage	THUMS v6.1
Annulus fibrosus – ground substance	L1-2: C1 = 0.37, C2 = 0.0925 L2-4: C1 = 0.27, C2 = 0.0675 L4-5: C1 = 0.19, C2 = 0.0475	1.0e−9	0.45	8-node, RI solid	Hyperelastic, Mooney-Rivlin	Schmidt et al. (2006) and Schmidt et al. (2007)
Annulus fibrosus – Collagen fibers	nonlinear stress- strain curves	–	–	truss	nonlinear stress- strain curves	Shirazi-Adl, Ahmed & Shrivastava (1986)
Nucleus pulposus	C1 = 0.12, C2 = 0.03	1.0e−9	0.4999	8-node, RI solid	Hyperelastic, Mooney-Rivlin	Schmidt et al. (2006) and Schmidt et al. (2007)
Ligaments	nonlinear stress- strain curves (Fig. 2)	1.0e−9	0.3	4-node, FI membrane	orthotropic, nonlinear stress -strain curves	Mattucci et al. (2012) and Östh et al. (2016)

### Ligaments

**Figure 2:**
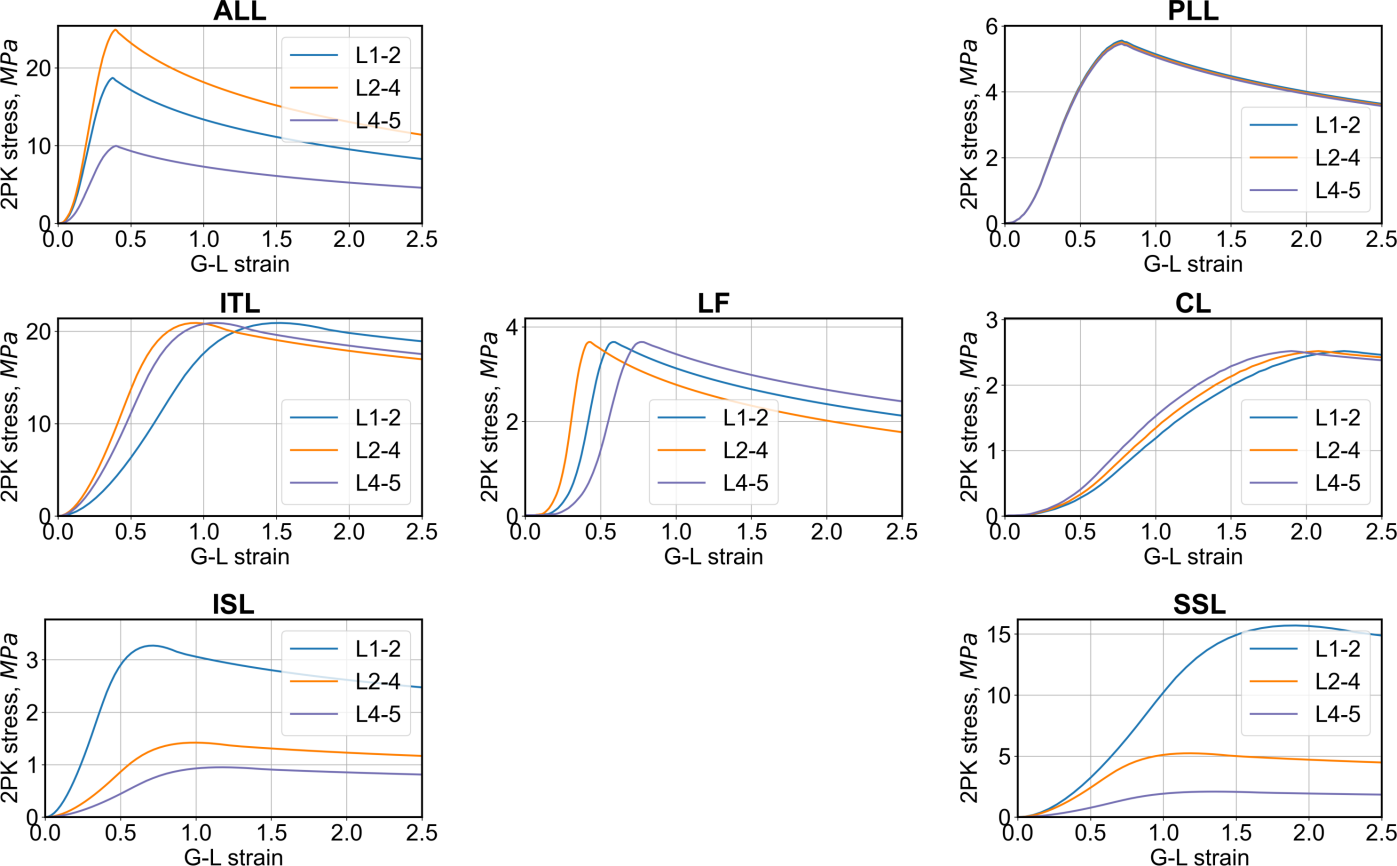
2nd Piola–Kirchhoff (2PK) stress–Green–Lagrange (G-L) strain curves from the current FE model for different ligament types.

In the lumbar spine of the THUMS, there were seven anatomical ligament types: anterior longitudinal ligament (ALL), posterior longitudinal ligament (PLL), intertransverse ligament (ITL), capsular ligament (CL), ligamentum flavum (LF), interspinous ligament (ISL), and supraspinous ligament (SSL), as shown in Fig. 1C. Ligaments in the THUMS model were represented using 4-node membrane elements and linear-elastic material properties (*MAT_FABRIC) with 50% damping. After modifications, soft tissues were modeled using large strain 4-node membrane elements with orthotropic material properties and nonlinear stress–strain behavior (*MAT_FABRIC), and a damping of 5% was introduced. The nonlinear curves were calibrated based on data from the literature (Mattucci et al., 2012; Östh et al., 2016), where the authors described simplified curves with corresponding characteristic points. The curves were extended beyond the initial range of strains to improve the stability of the numerical algorithm and prevent a sudden rupture. Such damage is unlikely in humans because ligaments are additionally connected to other soft tissues (muscles, discs, bones). Similar modeling was also used in the post-fracture behavior of the open human body model (HBM)-ViVA (Östh et al., 2016; Östh et al., 2017). For the THUMS model, the ligament thickness values were set constant for all soft tissues (1 mm). In the current model, the ligaments were divided into three groups: L1-2, L2-4, and L4-5, where modified values of thickness from a range of 0.5 mm to 5.0 mm were assumed (Table 2). The thickness of the ligaments was adjusted to values selected from a range obtained on the basis of articles (Chazal et al., 1985; Pintar et al., 1992). Stress–strain curves from the current FE model are presented in Fig. 2.

### Vertebrae

The geometrical and material properties of vertebrae in the modified lumbar spine of the THUMS remained unchanged compared to the original THUMS. Vertebrae were modeled by two bone types: cortical bone (*MAT_PLASTICITY_WITH_DAMAGE) and cancellous bone (*MAT_DAMAGE_2). The cortical bone was modeled by 4-node shell elements (*SECTION_SHELL) with full integration (FI). The thickness of those elements ranged from 1 mm at Th12 to 1.98 mm at L5. The cancellous bone was modeled by 8-node solid elements (*SECTION_SOLID) with reduced integration (RI) and nodal pressure averaging.

### Boundary conditions and validation tests

Boundary conditions were applied to both models through load tables that are depicted in Fig. 1A. The bottom load table was fixed, whereas the specific force, moment, or prescribed motion was applied to the top table. To simulate epoxy fixing, some nodes of vertebra Th12 and L5 were constrained to the tables using a constrained nodal rigid body (*CONSTRAINED_NODAL_RIGID_BODY). The compression load was applied using cable beam elements (*SECTION_BEAM_TITLE, *MAT_CABLE_DISCRETE_BEAM), and the load path was assumed following the literature (Renner et al., 2007).

**Table 2:** Thickness of ligaments of the lumbar spine model.

**Ligament**	**Thickness L1–2, mm**	**Thickness L2–4, mm**	**Thickness L4–5, mm**
Anterior Longitudinal Ligament (ALL)	2.5	3.5	1.5
Capsular Ligament (CL)	1.0	1.0	1.0
Ligamentum Flavum (LF)	5.0	5.0	5.0
Supraspinous Ligament (SSL)	4.0	3.0	2.0
Interspinous Ligament (ISL)	2.0	1.5	1.0
Intertransverse Ligament (ITL)	5.0	5.0	1.0
Posterior Longitudinal Ligament (PLL)	0.5	0.5	0.5

Sixteen experimental tests were selected for comparison with numerical results. For 13 tests, the ranges of motion (ROMs) were compared between simulations and experiments; for the remaining three tests, the intradiscal pressures (IDPs) were compared. All experimental tests are summarized in Tables 3 and 4, where references to experimental data are given. The experimental results of IDPs were obtained for a single disc sample of L1-2 and L3-4 sections (Brinckmann & Grootenboer, 1991). For the IVDs from L2-3 and L4-5, the experiments were conducted on 6 samples. In the fourth column of Table 4 (Compression Force, N), the compressive forces adopted for the tests are listed. Table 4 summarizes the list of remaining high-speed tension and shearing tests from the work by Demetropoulos et al. (1998). The tests of numbers 1, and 2 were selected for the model calibration; the remaining tests were selected for the model validation. The model was considered valid when the range of motion of the whole lumbar spine section was within the range of the corresponding experimental data.

**Table 3:** Quasi-static experimental tests for a range of motion (ROM) and interdiscal pressure (IDP).

**Test number**	**Test name**	**Test type**	**Compression Force, N**	**Moment, Nm**	**Reference**
1	Compression	ROM	1000	–	Renner et al. (2007)
10, 11, 12		IDP	300/1000/2000		Brinckmann & Grootenboer (1991)
2	Flexion	ROM	100	7.5	Xu et al. (2017)
3	Extension	ROM	100	7.5	Xu et al. (2017)
4	Lateral bending	ROM	100	7.5	Xu et al. (2017)
5	Torsion	ROM	100	7.5	Xu et al. (2017)
6	Flexion + Compression	ROM	1175	7.5	Xu et al. (2017) Renner et al. (2007)
7	Extension + Compression	ROM	500	7.5	Xu et al. (2017) Renner et al. (2007)
8	Lateral + Compression	ROM	700	7.8	Xu et al. (2017) Renner et al. (2007)
9	Torsion + Compression	ROM	720	5.5	Xu et al. (2017) Renner et al. (2007)

**Table 4:** Dynamic experimental tests for range of motion.

**Test number**	**Test name**	**Test type**	**Maximum displacement, mm**	**Reference**
13	Anterior shear	ROM	35	Demetropoulos et al. (1998)
14	Lateral shear		12	
15	Posterior shear		35	
16	Tension		2.5	

### Statistic metrics

In the field of curve analysis, assessing the similarities between curves is a fundamental step in many applications, such as biomedical research. However, identifying the best statistical metric to quantify the similarity between curves can be challenging and depends on various factors, such as data characteristics, noise level, and sampling frequency.

In this study, the similarities evaluation between sets of experimental and numerical curves for both tested models was made by employing three different statistical metrics, namely Pearson correlation coefficient, Strague-Geers MPC metrics (Ray, Anghileri & Mongiardini, 2008), and weighted integrated factor (WIFac) (Hovenga et al., 2005). Pearson correlation is one of the most widely used metrics in curve analysis and measures the degree of linear correlation between two sets of data. Pearson correlation is particularly useful when the data follows a normal distribution, and the relationship between the two sets of data is linear. Strague-Geers metrics is a non-parametric metric that determines the area between two curves. This metric is more robust to noise and outliers in the data and can provide more accurate results when the data is non-linear or non-uniformly sampled. Lastly, WIFac is a recently introduced metric that combines the advantages of both Pearson correlation and Strague-Geers metrics. WIFac is a robust and efficient metric that can handle noisy and irregularly sampled data. It is particularly useful in cases where the data contains outliers, and the relationship between the two sets of data is complex. By employing these three different metrics, a comprehensive analysis of the similarities between different sets of curves was made. The metrics were computed for each individual segment and subsequently averaged for the whole section. For the response corridors, the correlation was calculated separately for each of the curves that constitute a given experimental result and then averaged for the whole corridor. The final output represents the mean value for both the static and dynamic tests performed for the current and THUMS FE models.

## Results

This section presents the results of validation tests conducted for the two FE models: the current and THUMS v6.1. Both models were validated against experimental results obtained from the literature.

### Range of motion in quasi-static tests

**Figure 3:**
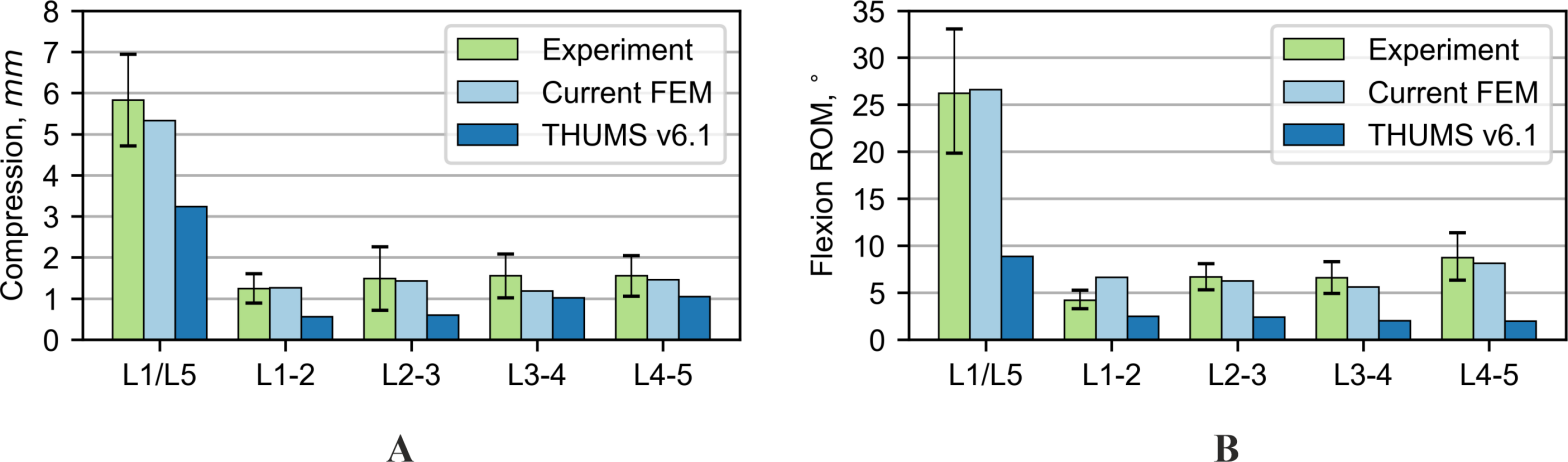
Comparison of range of motion results for various loading modes: (A) 1,000 N compression, (B) 7.5 Nm flexion bending.

The FE models were first subjected to compression with a force of 1,000 N. The shortening of the entire L-section and individual intervertebral discs (IVDs) is presented in Fig. 3A. The results for the current FE model are within the range of the experimental data on all levels and represent an improvement over the results for the original THUMS model. For the flexion test, the range of motion of the entire L-section (L1/L5) of the current FE model was inside the range of the experimental results (Fig. 3B). The rotations for some of the individual IVDs did not fit within the allowable range of motion. Only one segment L1-2 was out of the range of 1.6°. In the case of the THUMS model, these differences in individual vertebrae ranged from 0.8° to 4.2°.

**Figure 4:**
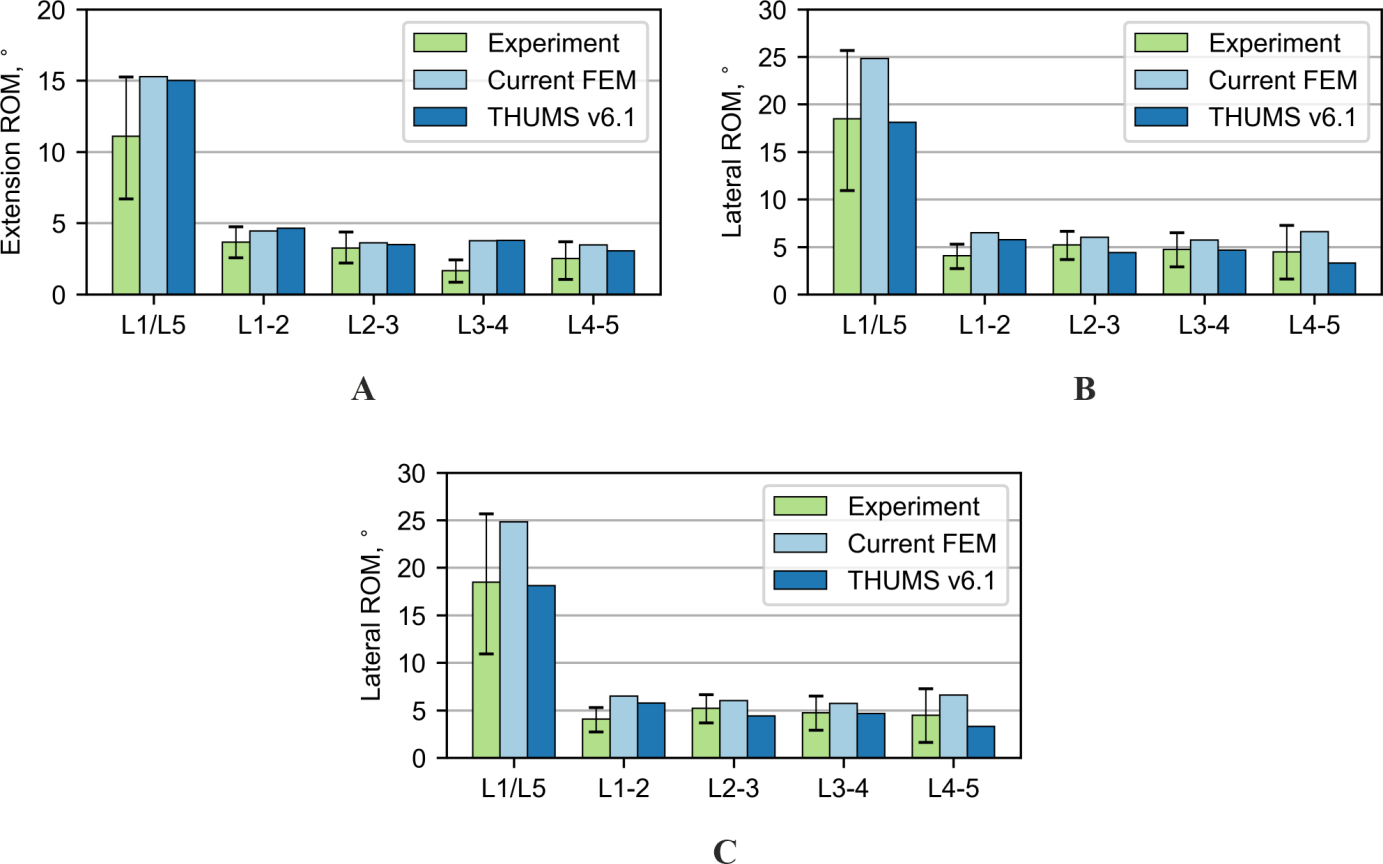
Comparison of range of motion results for various loading modes: (A) Nm extension, (B) 7.5 Nm lateral bending, (C) 7.5 Nm torsion.

Another test performed was the extension; its results are shown in Fig. 4A. The results of the current model and the THUMS model agree with most of the range of motion from the experiment. The biggest difference occurs on the L3-4 segment, 2.8° and 2.9° for the current and the THUMS model, respectively. Figure 4B presents the results for the lateral bending test. In the current model and the THUMS model, the difference was 0.6° and 0.1° for the L1-2 segment, respectively. For the torsion test (Fig. 4C), the results for both models mostly fell within the ranges of motion obtained from the experiment. In the current model, only the L4-5 segment slightly exceeded the upper limit by 0.36°.

### Range of motion in combined loading modes

**Figure 5:**
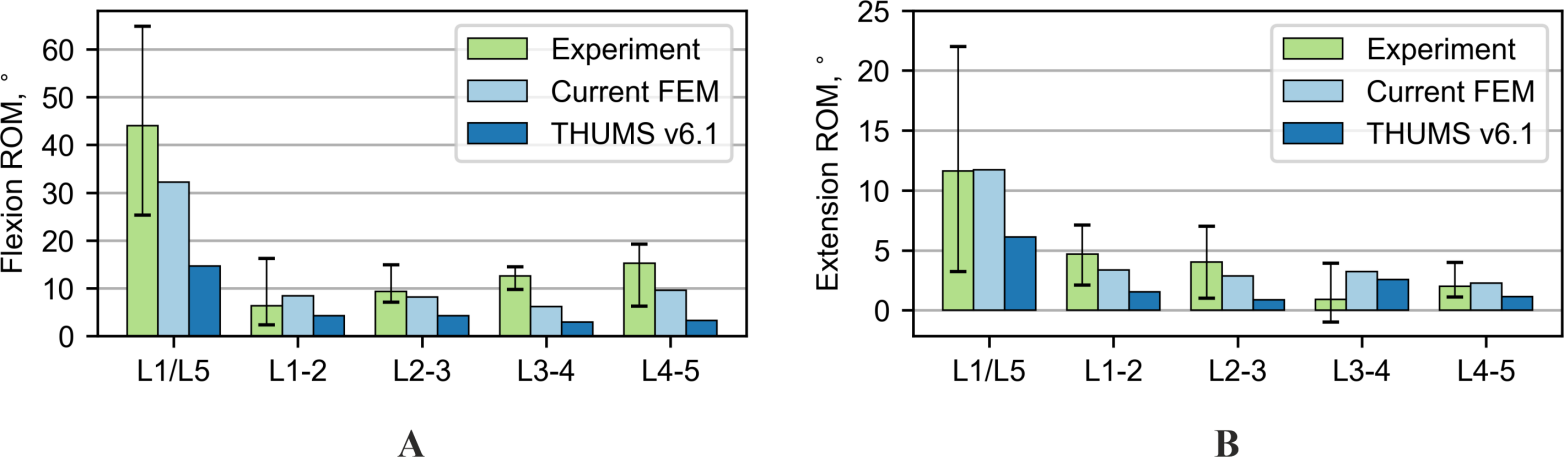
Comparison of range of motion results for various loading modes: (A) combined 1,175 N compression and 7.5 Nm flexion, (B) combined 500 N compression and 7.5 Nm extension.

The second group of tests consisted of four combined loading modes in which a compressive force was applied to the models simultaneously with a pure bending moment. In the first two tests, bending in the sagittal plane was analyzed. For a compression force of 1,175 N combined with a flexion moment of 7.5 Nm (see Fig. 5A), the rotations for the current model turned out to be consistent with the reference experimental values. Only the L3-4 segment failed 4.15° to fall within limits. In the case of the THUMS model, the rotations were outside the experimental data, where the differences ranged from 2.8° to 6.8°. For a compression force of 500 N combined with an extension moment of 7.5 Nm (see Fig. 5B), the current model fits very well with the experimental results. The THUMS model also achieved a good agreement with the results, only the response of L1-2 and L2-3 segments were too stiff, where the differences were 0.6° and 0.1°, respectively.

**Figure 6:**
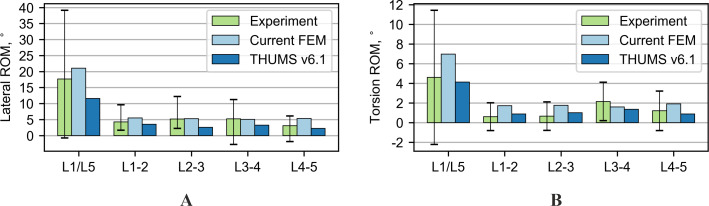
Comparison of range of motion results for various loading modes: (A) combined 700 N compression and 7.8 Nm lateral bending, (B) combined 720 N compression and 5.5 Nm torsion.

In the next performed tests, the combined loads acting in the coronal and transverse planes were analyzed. The results for these tests are summarized in Fig. 6. Both models successfully passed these tests, considering both the range of motion of the entire section and the ranges of motion for each functional spine unit (FSU).

**Figure 7:**
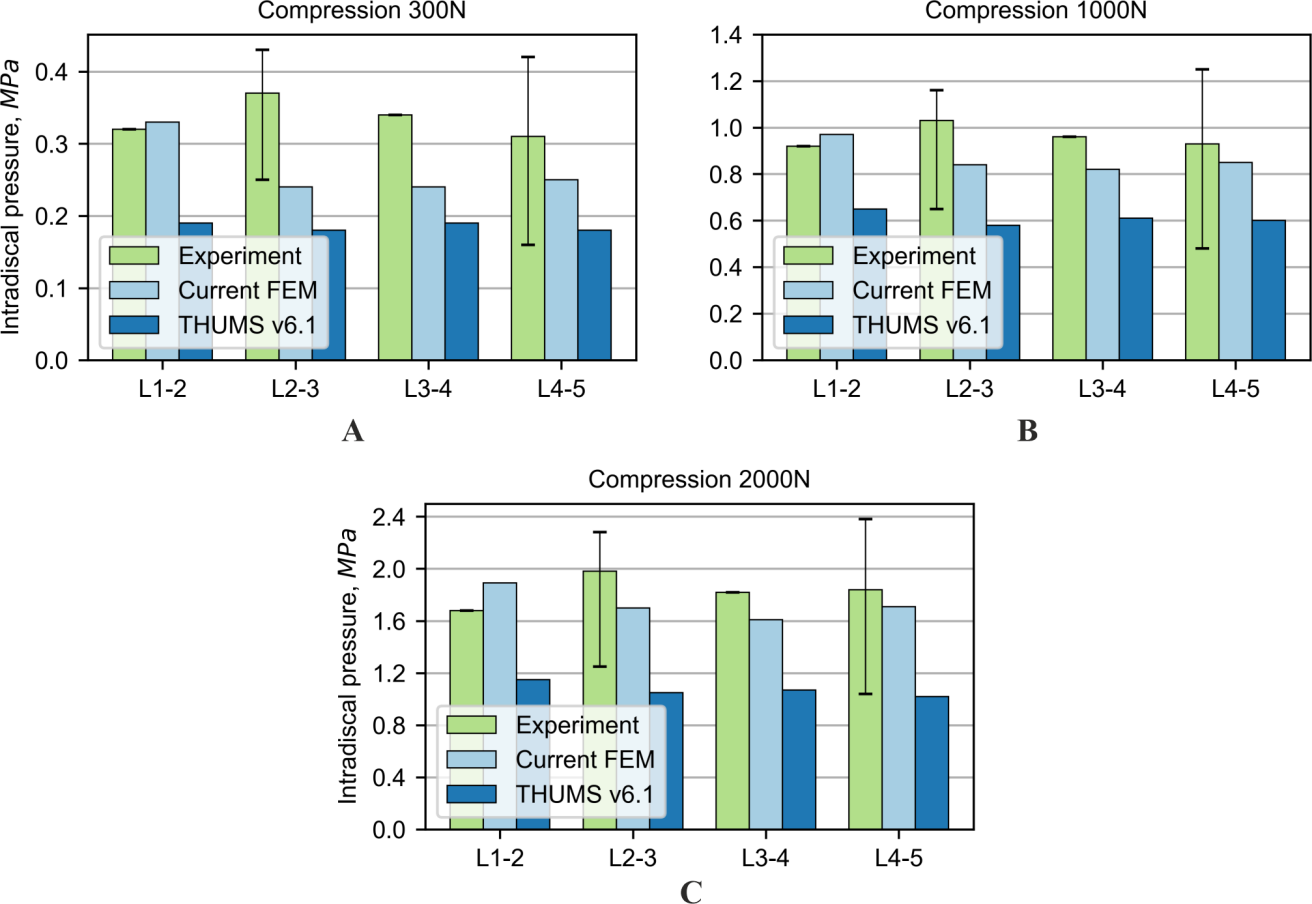
Comparison of intradiscal pressure values for compression force: (A) 300 N, (B) 1,000 N, (C) 2,000 N.

### Intradiscal pressures in compression

In addition to the previously described tests, the examination of the pressure acting in the nucleus pulposus was performed for the three different values of compression force. For the numerical simulations, the intradiscal pressure in the NP was determined as the mean of the pressure acting in all finite elements of the NP. The results for the current model are consistent with the reference experimental results; see Fig. 7. In the case of the THUMS model, the obtained results are not convergent with the experimental data and the current model.

### Range of motion in dynamic tests

**Figure 8:**
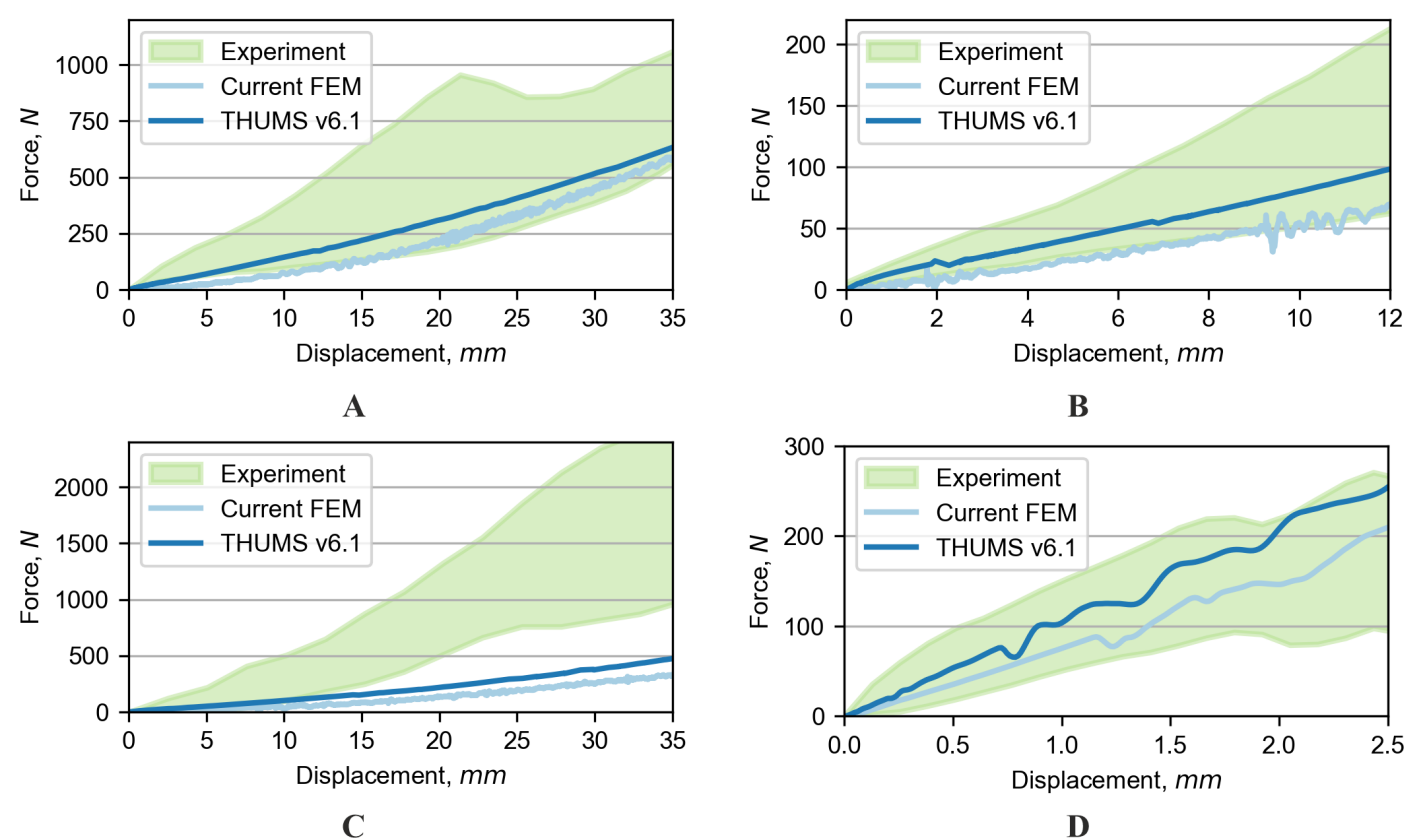
Comparison of range of motion for dynamic tests: (A) Anterior shear, (B) lateral shear, (C) posterior shear, and (D) tension.

The last validation tests which were conducted are the examination of the ROM in the tension and shear tests. The responses of experimental data, the THUMS model and the current model for shearing tests are presented in Figs. 8A–8C. Both numerical models were close to or within the response corridor of shearing tests in the anterior and lateral directions. For the posterior shearing test, however, both of the models appeared to be too compliant compared to the experimental results. In all of the cases of shearing tests, the THUMS model was stiffer compared to the current FE model. In the 250 N tension test (see Fig. 8D), the responses of both models were inside the response corridor acquired from the experiment.

### Statistic analysis

The statistical metrics comparing the similarities between experimental and numerical results for various loading conditions static (flexion, extension, lateral bending, and torsion) and dynamic (anterior, lateral, and posterior shear, and tension) at different spinal segments or for the whole segment were calculated. The overall average rating of Pearson correlation was 0.98 and 0.95 for the current model and the THUMS model, respectively. The Sprague Geers Comprehensive index for the current model was 42% and for the THUMS model it was 93%. The WIFac rating equaled 77% for the current model and 57% for the THUMS model. More detailed results are summarized in Table 5.

**Table 5:** Statistic metrics of the similarities between the experimental and numerical results.

**Loading**	**Section/Segment**	**Pearson**		**Sprague Geers Comp.**		**WIFac**	
		**Current**	**Thums**	**Current [%]**	**Thums [%]**	**Current [%]**	**Thums [%]**
Flexion	L1/L5	1.00	0.99	4	267	82	27
	L1/L2	1.00	1.00	7	103	69	47
	L2/L3	1.00	0.99	3	232	78	29
	L3/L4	1.00	0.99	4	301	70	24
	L4/L5	1.00	0.99	1	434	79	18
Extension	L1/L5	0.98	0.94	18	20	77	67
	L1/L2	0.96	0.86	8	17	79	58
	L2/L3	1.00	0.96	3	19	90	71
	L3/L4	0.99	0.98	44	38	54	53
	L4/L5	0.91	0.89	22	13	69	63
Lateral bending	L1/L5	0.99	0.97	13	51	91	68
	L1/L2	0.99	0.98	22	8	77	84
	L2/L3	0.98	0.97	15	53	83	63
	L3/L4	0.99	0.98	10	34	87	73
	L4/L5	0.99	0.94	7	112	90	44
Torsion	L1/L5	0.98	0.92	18	27	80	68
	L1/L2	0.80	0.66	23	24	72	52
	L2/L3	1.00	0.96	33	17	73	70
	L3/L4	0.98	0.97	4	13	91	71
	L4/L5	0.98	0.93	12	55	84	73
Static average		0.98	0.94	13	90	79	56
Anterior shear	L1/L5	0.96	0.98	69	38	71	83
Lateral shear	L1/L5	0.99	1.00	121	41	60	78
Posterior shear	L1/L5	0.99	0.99	566	320	21	33
Tension	L1/L5	0.96	0.96	7	34	79	69
Dynamic average		0.98	0.98	189	108	58	66
Average		0.98	0.95	42	93	77	57

## Discussion

In this article, a modified FE model of the lumbar spine based on the THUMS numerical model was validated against the experimental results of cadaveric tests available in the literature (Xu et al., 2017; Renner et al., 2007; Demetropoulos et al., 1998; Brinckmann & Grootenboer, 1991). The purpose of this study was to improve the response of the lumbar spine model in flexion by more realistic modelling of the soft tissues. An additional goal was to extend the validation of the THUMS model by testing the range of motion also in the combined loading modes and testing the pressure level in the discs. As a result, it is possible to investigate in detail deformation and the state of stresses in individual soft tissues. Numerical modeling of entire sections of the spine allows for analysis of risk injury that can occur in the human body during car accidents (Ivancic, 2013; Yoganandan et al., 2013; Pachocki et al., 2021; Kaufman et al., 2013).

Finite element models of the lumbar spine have been studied extensively using a variety of loading conditions, including compression loading. Compression loading can be used to study the behavior of the lumbar spine under loads that are similar to those typically encountered in daily activities. This can be important for understanding the effects of these loads on the spine and developing interventions to prevent or treat spinal disorders. A compressive force of 1,000 N was used for the calibration process, according to the experimental study. The current lumbar spine FE model presented a better response to this loading condition than the THUMS model.

Validation of lumbar spine FE models for tension and distraction loading conditions can be challenging. The lumbar spine is a complex structure, and these loading conditions can create complex stresses and strains within the spine that are difficult to measure experimentally. Additionally, the spine has a complex response under such loading, which is difficult to analyze in experimental tests fully. There have been several studies (Gay et al., 2008; Bae et al., 2020) that attempted to replicate the tension and distraction loading conditions on the lumbar spine using external loading devices, such as pull-tests on cadaveric spines, but these methods may also be insufficient to capture the true *in-vivo* response of the spine under tension/distraction. A literature review shows that there is a lack of tension validation for FE lumbar spine models (Xu et al., 2017). An experimental test by Demetropoulos et al. (1998) was done on cadaveric lumbar spine sections fixed at both ends and then tensioned. The same technique was performed for the current analysis. The simulation results for the modified model were consistent with the reported experimental data (within their range). Moreover, the obtained results were close to the average literature data. The THUMS model presented a stiffer response.

The predicted numerical results of the ROM analysis combining axial force and pure bending moments, matched with in vitro experimental data (Panjabi et al., 1994; Renner et al., 2007; Yoganandan et al., 1996). Additionally, a 1,175 N compressive force (Rohlmann et al., 2009) was used to simulate the model’s behavior during the carrying of external weight. Following *in vivo* experimental data from Rohlmann et al. (2001), a 100 N compressive follower force was added during the ROM in flexion, extension, and lateral bending motion. However, the compressive force leads to a significant ROM decrease for axial rotation. In extension, lateral bending, and axial rotation, the numerical results obtained for both numerical models were consistent with the experimental results. In all presented tests, the THUMS model predicted a slightly stiffer response. In addition, the lumbar spine response to complex load states also compares very well with the experimental ranges provided by the literature for the current model. However, the original model of THUMS v6.1 did not meet the condition of the range of experimental results for different loading modes of compression and flexion. In summary, these results motivated the authors to modify the THUMS model.

IDP validation was relatively more difficult due to the limited number of experimental studies available in the literature. Rohlmann et al. (2001) conducted *in vitro* research and determined the IDP in whole lumbar spine samples (L1–L5) by applying pure moments (axial rotation, extension, flexion, and lateral bending) plus a 280 N follower load. Nonetheless, the authors found that the IDP was smaller for the flexion compared to the extension for the same loading conditions, contradicting the results obtained by Wilke et al. (2008), Heuer et al. (2008) and Dreischarf et al. (2014) in the FE study of eight sections of the lumbar spine. *In vitro* experiments were also conducted on FSU samples, which contain one intervertebral disc and two vertebrae of the lumbar spine section (Heuer et al., 2008; Wilke et al., 2008). However, the IDP obtained for the whole section of the lumbar spine sample (Rohlmann et al., 2001) and for FSU specimens (Heuer et al., 2008; Wilke et al., 2008) differ significantly. For example, mean IDP in flexion (7.5 Nm) measured in L4-5 for the whole lumbar section (Rohlmann et al., 2001) and for FSU (Wilke et al., 2008) were 0.8 MPa and 0.4 MPa, respectively. The authors decided to validate the current FE model of the lumbar spine using results of in vitro experiments of discs conducted by Brinckmann & Grootenboer (1991). The current model predicted the intradiscal pressure in the NP very well for each functional spine unit and loading state. However, the pressure determined in the THUMS model was out of the range of the experimental data. This comparison indicates, that the current model improved the prediction of the experimental results. It is worth mentioning that the literature review indicates limited experimental data in the area of intradiscal pressure for NP.

In this study, the evaluation of the similarities metrics between experimental and numerical curves for both current and THUMS models were made. The study employed three statistical metrics. The results showed that in selected validation cases the current FE model performed better compared to the original THUMS v6.1 L-spine model in terms of selected similarity metrics, *i.e.*, Pearson correlation, Sprague-Geers Comprehensive index, and WIFac rating. The result of a Pearson correlation indicates a better linear relationship with experimental data for the current model than the THUMS model. Sprague-Geers indices indicated that the results of the current model were closer to experimental data, in terms of magnitude and phase, compared to the THUMS model. Lastly, the WIFac showed that the overall shape of simulation curves in current model was closer to the expermintal results. By employing these three different metrics, the study provided a comprehensive analysis of the similarities between different sets of curves. Usage of these metrics allowed the evaluation of the relationships between curves with varying degrees of non-linearity and sampling frequency. Additionally, the study’s approach to averaging the metrics for each individual segment and subsequently for the whole section and corridors provides a more accurate representation of the similarities between the curves. Finally, the average value for all static and dynamic tests was shown. These results suggest that the current model may be more suitable for numerical analysis in biomedical research applications.

In numerical modeling, an important issue is the selection of a material law that best reflects the mechanical response of soft tissue. It is possible to describe the behavior of the structures in the lumbar spine section using various hyperelastic and other nonlinear material models such as Neo-Hookean, Mooney-Rivlin, or Yeoh models. Nevertheless, it is important to validate the kinematics of the spinal section as well as the mechanical response (pressure and forces) (Dreischarf et al., 2011). Dreischarf et al. (2014) presented different FE models subjected to pure compression and moments. They recommended analysis of the combined load of compression-torsion in experimental research. Hence, according to those recommendations, this article presents a wide range of loading modes acting on the lumbar spine, as well as an analysis of the pressure acting on the NP. In addition, a validation of tensile forces acting in the lumbar section was performed, which is not usually conducted. However, these loading modes are frequently observed during road accidents (Pachocki et al., 2021). The original contribution of the article is a separation of the NP from the surrounding bones and the AF. This has an impact on the response of this structure during different loading modes. The NP does not participate in the transmission of the tensile load, but in compression it does. Furthermore, the current model differs from the original THUMS model not only in constitutive laws and some values of material properties but also in the content and distribution of collagenous fibers in the AF ground substance and the anatomical thickness of ligaments. The described changes and modifications of the model resulted in a significant improvement in the model’s response to the given forces and moments during validation.

Numerical models are very useful in biomedical engineering, *e.g.*, as a tool for the examination of risk analysis during car accidents. The differences in the results between the modified model and the original THUMS model show that one must be careful in drawing biomechanical conclusions from only one FE model for a particular population. The model needs to be validated to make more accurate analyses. Although the model may work well for examining the general response, it may not be suitable for detailed analyses. Nevertheless, the experimental analysis is still insufficient to explain the detailed failure mechanism, such as failure morphology and functions of soft tissues in the lumbar spine. Moreover, the anatomical and geometrical properties differ for experimentally tested samples (Mattucci et al., 2012), which may account for the differences in the obtained numerical results. The sensitivity of these parameters can influence the kinematic and mechanical response of the structure (Meijer et al., 2011; Robin, Skalli & Lavaste, 1994; Natarajan & Andersson, 1999; Lu, Hutton & Gharpuray, 1996; Niemeyer, Wilke & Schmidt, 2012). Moreover, the complex interactions and combinations of several geometrical and material parameters can govern the behavior of a model under particular combined loads. Hence, it is important to further develop comprehensive FE HBMs.

The presented work has its limitations:

• The 50th percentile of the AM THUMS v6.1 model was used in the analysis. Considering the biological biodiversity of the human population, the model yields results that properly describe the behavior of average male subjects. The current model can be used to investigate the biomechanics of a specific population. However, sensitivity analysis could provide importance for the development of numerical studies on the response of the lumbar spine section. It would show what structures, geometries, or material descriptions play a crucial role in the transmission of loads to the lumbar section.
• The presented validation was performed on the basis of the experimental data selected by the authors. However, more experimental data are available in the literature (Guan et al., 2007; Niosi et al., 2008; Pearcy, 1985; Yamamoto et al., 1989) that can be used in validation. The authors selected experimental studies conducted for a male subject due to the tested male model of the lumbar spine. However, due to the biodiversity of the population, it would be necessary to verify and validate the obtained data with other experimental works.
• A comprehensive analysis of the range of motion of the model in the sub-injurious range is an important first step in the validation of the model that may be used in crash analysis. However, it would be necessary to examine the model in conditions similar to those of a road accident, namely high strain rates, compression force, and moments (Minster, Lafon & Beillas, 2023; Yoganandan et al., 2023; Tushak et al., 2022; Tushak et al., 2023). The current validation set is not sufficient to fully evaluate the model’s ability to accurately predict the outcomes of a car crash.

## Conclusions

A numerical model of the lumbar spine section was created based on the data available in the literature. The model passed most of the validation tests for various load combinations. The modifications introduced to the model resulted in a better agreement of the results with experimental data than for the original THUMS model. The separation of the NP from the AF and the surrounding bones changed the response of this structure to the action of compressive and tensile forces and allowed for obtaining the mechanical pressure in accordance with the literature. Moreover, the current model was validated against the kinematics of the spinal section, as well as the mechanical response. Considering the extensive complexity of this model, the level of this validation can be considered satisfactory. In the future, it is possible to expand the validation set with more extreme load cases.

## Additional Information and Declaration

### Data Deposition

The following information was supplied regarding data availability: The data is available at Most Wiedzy: Wiczenbach, T., Pachocki, Łukasz, Daszkiewicz, K., Łuczkiewicz, P., & Witkowski, W. (2023). Validation of lumbar spine finite element model [Data set]. Gdańsk University of Technology. https://doi.org/10.34808/4n5h-3949.
